# Does ‘heads-up’ cardiopulmonary resuscitation improve outcomes for patients in out-of-hospital cardiac arrest? A systematic review

**DOI:** 10.29045/14784726.2020.12.4.4.16

**Published:** 2020-03-01

**Authors:** Andrew Elphinstone, Samantha Laws

**Affiliations:** University of London; University of London

**Keywords:** cardiopulmonary resuscitation, heads-up positioning, out-of-hospital cardiac arrest

## Abstract

**Introduction:**

Survival rates for patients in out-of-hospital cardiac arrest have remained around 10% in the United Kingdom for the past seven years. If outcomes are to be improved, research into new methods of advanced life support is required. One such method may be ‘heads-up’ cardiopulmonary resuscitation.

**Methods:**

A systematic review of literature exploring heads-up cardiopulmonary resuscitation was conducted in an attempt to identify its effects on survival to discharge and neurological outcome.

**Results:**

A comprehensive search of CINAHL, MEDLINE and Google Scholar was undertaken. Six papers were classed as sufficiently relevant for inclusion. Included studies were generally of low quality and none studied the effect of heads-up cardiopulmonary resuscitation on out-of-hospital cardiac arrest patients. Animal studies identified a significant reduction in intracranial pressure and increase in cerebral and coronary perfusion pressure for use of augmented heads-up cardiopulmonary resuscitation in the porcine model of cardiac arrest.

**Conclusion:**

Further research is required to analyse the effects and potential benefits of augmented heads-up cardiopulmonary resuscitation in out-of-hospital cardiac arrest.

## Introduction

Paramedics in the United Kingdom have been trained in the delivery of advanced life support (ALS) since 1979, with techniques being refined and updated over time in an attempt to improve survival to discharge for out-of-hospital cardiac arrest (OHCA) patients (College of Paramedics, 2015). Despite these changes, data from NHS England (2018) show that in August of that year, the survival to discharge of all-cause OHCA patients treated by UK ambulance services was 10.4%, with even fewer than this discharged with favourable neurological outcome (exact percentage unlisted within these statistics). The earliest available comparable statistics from NHS England (2011) show OHCA survival to discharge rates have remained largely unchanged over the last seven years, and it is clear that more research is needed to identify areas of improvement for treatment of OHCA by ambulance services.

A prospective one-month meta-analysis of 27 European countries by Gräsner et al. (2016) identified a number of countries with better survival to discharge rates than the United Kingdom, meaning that there are current ongoing techniques and systems in place that UK ambulance services either need to start or need to do more effectively. Two such areas may be increased public education in cardiopulmonary resuscitation (CPR) and publicly accessible defibrillators (Bækgaard, Viereck, Møller, Ersbøll, & Lippert, 2017; Song et al., 2018), both of which require large systematic changes in public education through collaboration between emergency services and local government services. However, this systematic review aims to identify a novel approach to CPR that emergency medical services (EMS) in Florida, United States, have reportedly implemented to good effect.

Pepe et al. (2016) delivered a seminar at a conference describing a resuscitation technique known as ‘heads-up’ CPR, described as a process by which a patient in OHCA in placed on an orthopaedic scoop elevated at the head end by approximately 30° by being placed on a large pelican case. This process is accompanied by intubating the patient, affixing an impedance threshold device (ITD) to the end of the endotracheal tube and delivering chest compressions through an active compression–decompression (ACD) device that implements active chest recoil. Pepe et al. (2016) explain that this process decreases intracranial and intrathoracic pressures, thereby increasing cerebral and coronary perfusion pressure, and are claiming an increase in survival of all-cause OHCA from 17.4% to 36% across 2014–2015. However, this information is provided in abstract only and no subsequent data have been released disclosing further information on this doubling of survival rates; neither has ‘increased survival’ been given a more exact definition, nor is ‘all cause’ OHCA specified to include traumatic cardiac arrest. NHS England (2018) measure success rates in OHCA by ‘survival to discharge from hospital’ and measure the quality of discharge by neurological outcome. While this abstract offers little in terms of tangible research or evidence to critique, the possibility of doubling survival rates in OHCA patients is certainly worthy of further investigation.

This systematic review sets out to determine if heads-up CPR (HUPCPR) improves patient survival to discharge and neurological outcomes in OHCA by critically analysing the supporting literature of the HUPCPR technique.

### Aims

To discern if HUPCPR improves patient survival to discharge for OHCA and improves patient neurological outcomes compared to supine position CPR.

### Objectives

- To explore whether HUPCPR improves patient survival to discharge compared to supine position CPR.
- To explore whether HUPCPR improves neurological outcome for survivors of OHCA compared to supine position CPR.
- To explore whether HUPCPR can be recommended for UK paramedic practice.

## Methods

A pilot search was conducted of Google Scholar based upon the PICO criteria exploring the available literature around HUPCPR:

- Population: Adult OHCA patients.
- Intervention: ‘Heads-up’ elevated cardiopulmonary resuscitation.
- Comparison: Standard supine CPR.
- Outcome: Identification of improved patient survival to discharge with particular focus on improved neurological outcome for patients.

The pilot search revealed one abstract of a conference by Pepe et al. (2016), which discussed a pre-hospital HUPCPR policy, four links to articles written on American EMS websites discussing the work by Pepe et al. (2016) and a link to a PowerPoint presentation by Lurie (2018) outlining the aetiology of HUPCPR and a brief insight into improved survival for OHCA patients. Several of the pieces discovered in the pilot search referenced animal studies into HUPCPR, while none alluded to any human based OHCA trials. Since the aforementioned abstracts, website articles and PowerPoint offer few to no data to critique, the inclusion criteria was expanded to include animal studies, as were referenced in some of the pilot study articles.

Therefore, the final inclusion and exclusion criteria consisted of:

- Inclusion criteria:
  - Studies monitoring the effect of heads-up positioning on outcome of cardiac arrest (with particular interest to survival with neurological outcome).
  - Human OR animal model of cardiac arrest studies.
  - Human OR animal cadaver model of cardiac arrest studies.
- Exclusion criteria:
  - Full text not available.
  - Foreign language paper with no available translation.

A systematic search of literature from the past 10 years from the databases AMED, CINAHL Plus and PubMed was conducted from 15 January to 19 February 2019. The search terms used were “heads up” or “elevat*” (a truncated search term) or “patient positioning” and “cardiopulmonary resuscitation”. The PRISMA reporting method was used (Moher, Liberati, Tetzlaff, Altman, & PRISMA Group, 2009; Supplementary 1).

### Critical appraisal

The primary author used the NICE appraisal checklist for quantitative intervention studies (National Institute for Health and Care Excellence, 2012) as it was deemed the most appropriate with which to critique the literature.

## Results

**Figure 1:**
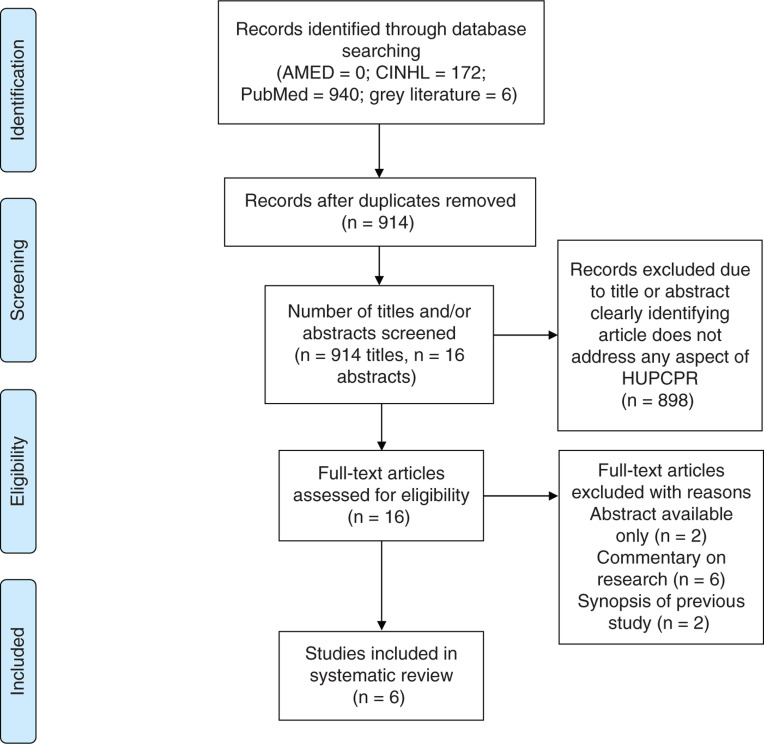
Figure 1. PRISMA flow diagram (Moher et al., 2009).

After removal of duplicates, 914 papers were screened, with 16 papers put forward for full-text review; this process is described in the PRISMA diagram (Figure 1) (Moher et al., 2009). After exclusion of 10 papers, six were included in this review (Table 1).

**Table 1.** Summary of included studies.

Authors (date)	Study aim and design	Outcomes measured	Key results	Critical appraisal summary
Debaty et al. (2015)	Examines potential benefits of HUT positioning compared to SUP and HDT positioning during CPR respectively. Twenty-two female Yorkshire pigs were sedated, intubated, ventilated and secured on a table designed to tilt the whole body according to the position required. VF was induced and left untreated for 6 mins prior to interventions outlined in the adjacent protocols. CPR was implemented with an ACD device that pulls the chest wall up after each compression rather than allowing passive recoil, and an ITD that lowers intrathoracic pressure during the decompression phase, thereby improving blood return to the heart.	Protocol A: To observe how CPR augmented with ACD and an ITD affects CoPP and CerPP comparatively as the patient changes position: SUP, HUT +30° or HDT -30° receiving 5 mins of ACD+ITD CPR in each position. Secondary objective of protocol A: To determine the importance of ITD by removing it while continuing CPR. Fourteen pigs examined. Protocol B: Eight pigs randomly selected from Protocol A. Blood flow to heart and brain measured via four injected microspheres into the LV; the first to gain baseline information 5 mins prior to induced VF, 4 mins after ACD+ITD CPR initiated, 1 min after HUT and HDT respectively. Protocol C: Eight pigs randomly selected from Protocol A. Observe impact that increasing levels of heads-up ACD+ITD CPR from +0° to +50° in 10° increments has on CoPP and CerPP.	Protocol A: CerPP increased with HUT (p < 0.05). No definitive increase in CoPP. Secondary outcome observed immediate drop in SBP, DBP and CoPP. Protocol B: Brain blood-flow 42% higher in HUT compared with SUP (decrease by 26% in HDT). No statistically significant difference in blood flow to heart across positions. Protocol C: Linear decrease in ICP and increase in CerPP as HUT angle increased (p < 0.001 for both values respectively). CoPP remained constant throughout positional changes.	Findings are generalisable to source populations.
Ryu et al. (2016)	Observes if head and thorax elevation has similar effect on systemic haemodynamics during CPR as previous study by Debaty et al. (2015) involving full body-tilt. Thirty female Yorkshire pigs. VF induced and left untreated for 8 mins prior to interventions outlined in the two study groups adjacent. Subjects were randomised into the groups outlined in the following column.	CerPP and CoPP were the primary outcomes measured for each group. Group A: 14 pigs in SUP experienced 2 mins of C-CPR where chest compressions were delivered via a piston device and allowed passive chest recoil. Subjects were then randomised to either 30° HUP or SUP without interruption in C-CPR for 20 mins. Group B: Sixteen pigs with resuscitation cycle and randomisation of positions identical to Group A. ACD+ITD CPR used as opposed to C-CPR in A. After 22 minutes of CPR, subjects were defibrillated with up to three 275J biphasic shocks. If ROSC was not achieved, 0.5 mg adrenaline and 25 mg amiodarone were administered. If ROSC was not achieved, CPR resumed with defibrillation every 2 mins and 0.5 mg adrenaline every 4 mins for a total of up to 15 mins. If ROSC was not achieved CPR was stopped. If ROSC was achieved subjects were then euthanised.	Group A: CerPP was significantly higher (p = 0.016) in HUP group. CoPP was not significantly different. Group B: CerPP was significantly higher in HUP group (p = 0.006). CoPP was not significantly different. Mean CerPP in ACD+ITD HUPCPR group was far higher than all other groups (p < 0.0001). No subjects in group A were resuscitated; 8/16 subjects from group B achieved ROSC, six from each sub-group.	Findings are generalisable to source population, but conclusion of evidence suggesting improved neurological outcome is unfounded as neurological outcome was not a measured outcome and therefore has been extrapolated from improved cerebral perfusion.
Kim et al. (2017)	Assesses ideal angle of tilt for optimal CerPP and CoPP using a whole-body tilt table. Twelve female farm pigs underwent similar procedural preparation to previous studies (change only in sedative administered). Subjects were left untreated in VF for 6 mins followed by 3 mins of ACD+ITD CPR prior to protocol implementation.	CerPP and CoPP measured at HUT of +30°, +45° or +60°, SUP and HDT of -30°, -45° or -60°. Four pigs were randomly assigned to each angle of tilt in two groups: Group 1: 5 mins HUT, 5 mins SUP, 5 mins HDT. Group 2: 5 minutes HDT, 5 mins SUP, 5 mins HUT. After a total of 18 mins of ACD+ITD CPR, subjects were administered defibrillated with a 200J biphasic shock.	Peak CoPP was found at +30°. Peak CerPP was found at +45° and +60°. CerPP increased significantly between each change in position from -60° to +45° across the angles tested (p < 0.001 for each respective change). CoPP increased significantly between each change in position from -30° to +45° across the angles tested (p < 0.001 for each respective pchange). All animals achieved ROSC post-defibrillation.	Findings are generalisable to the source population.
Moore et al. (2017)	Compares brain blood-flow between HUP and SUP positioning during a prolonged resuscitation effort. Eighteen female Yorkshire pigs underwent identical preparation as outlined in the first two studies. VF and left untreated for 8 mins, followed by 2 mins of SUP ACD+ITD CPR. Brain blood-flow was measured by microspheres injected into the LV.	Subjects were randomised into either SUP or HUP for 18 mins of continuous ACD+ITD CPR. Primary outcome measured was blood flow to the brain after 15 mins of CPR. Secondary outcomes measured after 5 mins of CPR was blood flow to the brain, ICP and end tidal CO_2_ after up to 20 mins of CPR. After 19 mins of CPR, 0.5 mg adrenaline and 25 mg amiodarone were administered, and defibrillated with 200J 1 min later. If ROSC was not achieved, CPR was continued, with defibrillation every 2 mins and 0.5 mg adrenaline administered every 4 mins. After the third shock, resuscitation was terminated if ROSC was not achieved. If ROSC was achieved, subjects were then euthanised.	Primary outcome: Brain blood-flow was 25% of pre-VF baselines rate in SUP group compared to 50% in HUP group. Secondary outcome: Blood flow was only slightly higher in HUP group. ICP remained fairly constant throughout the study for SUP group, and steadily declined in HUP (p ≤ 0.001 after 15 mins of CPR). End tidal CO_2_ gradually reduced at an equal rate in both groups. Five of eight pigs were successfully resuscitated from the HUP group compared to three of 10 for the SUP group.	Results are generalisable to source population. Significant flaw in that in one HUP group, subject results were not included in analysis without explanation; potential for bias if exclusion was deliberate due to poor results conflicting with preferred outcome.
Moore et al. (2018)	Tests hypothesis that similar changes in systemic haemodynamics would be witnessed across porcine models, PC models and HC models when comparing SUP and HUPCPR. The HCs were prepared within 24–48 hrs post-mortem in preparation for the study which occurred at a later date.	CerPP and mean systolic and diastolic ICP were continuously monitored in each of the three groups. Nine pigs were prepared identically as in the articles discussed previously; VF was induced and left untreated for 6 mins before initiating CPR for 2-min epochs in the following sequence: C-CPR, SUP ACD+ITD CPR and finally HUP ACD+ITD CPR. They were then defibrillated with up to three 200J biphasic shocks. If ROSC was not achieved, CPR was continued and 0.5 mg of adrenaline and 25 mg of amiodarone were administered IV until ROSC. Subjects were then euthanised. Three hours later the PCs were exsanguinated and filled with heparinised saline. The above epochs were then repeated. The HCs underwent 1-min epochs of the same sequence.	Consistent increase in CerPP and decrease in ICP across the three cardiac arrest models (p = 0.007 for all values when comparing HUP with SUP ACD+ITD CPR). Little or no change was witnessed in the HCs when comparing CerPP and ICP between C-CPR and SUP ACD+ITD CPR.	Findings are not generalisable to source population due to significant flaw in study design of HCs (explained in more detail in discussion).
Putzer et al. (2018)	Aims to determine the effects of HUP vs. SUP CPR on cerebral oxygenation and metabolism. Twenty pigs were prepared to allow study outcomes to be measured. VF was induced and left untreated for 8 mins. Pigs were then randomised to either HUP or SUP. CPR was commenced, simulating BLS by a mechanical device allowing passive chest recoil.	MAP, ICP (and consequently CerPP), cerebral regional oxygenation (rSO_2_), brain tissue partial oxygen pressure (P_bt_O_2_) and cerebral venous oxygenation (S_cv_O_2_) at 5-min intervals from 0 to 20 mins of CPR.	MAP increased after 5 mins of CPR in both groups, slightly favouring HUP. ICP increased significantly in SUP, but remained largely unchanged in HUP group. CerPP was significantly higher in HUP group during CPR throughout the study. rSO_2_, P_bt_O_2_ and S_cv_O_2_ were virtually identical in both groups during CPR.	Findings are generalisable to source population with minimal source of bias and robust methodology.

Note: ACD = Active Compression–Decompression; BLS = Basic Life Support; C-CPR = Conventional Automated Cardiopulmonary Resuscitation; CerPP = Cerebral Perfusion Pressure; CPR = Cardiopulmonary Resuscitation; CoPP = Coronary Perfusion Pressure; DBP = Diastolic Blood Pressure; HC = Human Cadaver; HDT = Heads-Down Tilt; HUP = Heads-Up; HUT = Heads-Up Tilt; ICP = Intracranial Pressure; ITD = Impedance Threshold Device; LV = Left Cardiac Ventricle; MAP = Mean Arterial Pressure; PC = Pig Cadaver; ROSC = Return of Spontaneous Circulation; SBP = Systolic Blood Pressure; SUP = Supine Position; VF = Ventricular Fibrillation.

### Description of included studies

The included studies for this review were of a lower level than anticipated (Oxford Centre for Evidence-Based Medicine, 2011); rather than pre-hospital focused randomised controlled trials, only porcine animal studies investigating HUPCPR were found as a result of the literature search.

The first five papers listed in Table 1 share conflicts of interest: author Lurie is inventor of the ITD and ACD and is consultant to the company that authors Metzger and Lick are employed by. Funding for the research was also provided by the same company and the repeated involvement of the aforementioned authors across these studies may have biased the validity of results obtained.

All six of the papers clearly addressed a focused issue, with virtually identical study protocols observed across the first four. Sample sizes, while small at face value, were deemed appropriate by all investigators when assuming an alpha level of 0.05 to reject the null hypothesis and a power of 95%. Samples were increased to accommodate for potential drop-outs in experimental procedure (due to dislodged monitoring equipment as in one pig studied by Putzer et al., 2018).

The investigative processes described in five of the papers (excluding Moore et al., 2018) required an element of randomised selection, which was largely well documented. However, Debaty et al. (2015) constructed three investigation protocols (see Table 1) for which the recruitment process was not described. This poor methodological recording is unlikely to have impacted upon the results in group A, as each subject experienced heads-up tilt (HUT), supine (SUP) and heads-down tilt (HDT) positioning, but may bias results within groups B and C, where eight pigs per group were selected for further testing. Ryu et al. (2016) similarly did not describe the randomisation process for the two groups observed within their study. These errors potentially bias results, with no indication given as to whether the randomisation process was blinded, potentially introducing selection bias. This dilutes the validity of these two papers with respect to answering the research question.

Each study measured outcomes in a largely similar way, yet due to the nature of tilting the patient, thereby altering arterial pressures at which the probes are sited, cerebral perfusion pressure (CerPP) calculations can vary significantly in HUP positioning if mean arterial pressure (MAP) is measured at the foramen of Monro rather than the right cardiac atria, and experts have yet to agree on which site is optimal (McNett et al., 2017). While it is unknown which method is more reliable, measurement at the right atria is more likely to be achievable in the pre-hospital environment. Furthermore, should it be established that measuring MAP is more accurate at the foramen of Monro, McNett et al. (2017) state that the estimated difference may be that of 15 mmHg less at the atria – this can be easily remedied mathematically by allowing for an overestimation of the same value when analysing results.

Methods for making these investigations’ results translatable to OHCA are limited, as acknowledged in each of the papers, due to their nature as animal studies. The authors of each paper acknowledged this by leaving study subjects untreated in ventricular fibrillation (VF) for 6–8 minutes prior to commencing control interventions – a reasonably accurate representation of OHCA, making results more transferable to paramedic practice. Moore et al. (2018) also identified that the lower limbs of a pig are far smaller proportionally than those of humans, and therefore created a translational human cadaver (HC) study (described more fully in Table 1) to make results more relatable to human OHCA. However, detrimentally and without explanation, catheters were inserted into the HC’s renal arteries to restrict flow of saline (blood replacement in the cadaver cardiac arrest model) to the extremities, therefore restoring the previously identified discrepancy between subject and target patient anatomy. Moreover, using saline as a substitute for blood further separates results from application to OHCA patients as it increases systemic flow that is unlikely to be present in cardiac arrest, either due to thromboembolic cause of arrest or ongoing coagulation due to reduced blood flow. Therefore this paper cannot meaningfully contribute to this review’s conclusion. As such, it cannot meaningfully contribute to this review’s conclusion. However, despite its flawed methodology, the paper remains included within this review for the reasoning that such a novel approach, with regards to bridging the gap between the porcine and human model of cardiac arrest, may be vital if repeated with more rigorous study technique in identifying the potential benefit of HUPCPR in OHCA.

Follow-up of subjects was short and for reasons undisclosed (either study design oversight or ethical granting) subject follow-up never progressed beyond return of spontaneous circulation (ROSC), when subjects were then euthanised. Therefore, unless ethics permit extended observational periods to assess neurological status, conclusions made by Ryu et al. (2016) cannot be extended to simulated survival to discharge for OHCA patients in this porcine model of cardiac arrest.

## Discussion

The results of the critical appraisal of literature are congruent, illustrating an emergent school of thought that ‘heads-up’ positioning during the porcine model of cardiac arrest reduces intracranial pressure (ICP) and increases CerPP. This does not answer the question of this systematic review, but may provide impetus for future research into the potential benefit of HUPCPR on survival of OHCA patients and neurological outcome.

Putzer et al. (2018) identified that HUPCPR alone, while statistically significantly reducing ICP and increasing CerPP, did not increase cerebral tissue oxygenation. Debaty et al. (2015) and Ryu et al. (2016) also previously demonstrated that CerPP values were significantly lower when comparing C-CPR to ACD+ITD HUPCPR. On the basis of these three papers, it is reasonable to conclude that HUPCPR without augmentation of ACD+ITD would unlikely be of benefit in OHCA patients, although further research in human cardiac arrest would be required to form a true conclusion.

Patients in cardiac arrest lose haemodynamic autoregulation, commonly causing cerebral oedema (Brule, Hoeven, & Hoedemaekers, 2018), resulting in raised ICP, reducing CerPP (as CerPP = MAP-ICP) and thus inducing hypoxic brain injury. Management of ROSC patients in hospital involves close monitoring and pharmaceutical control of their ICP to nullify this pathology (Malaguit et al., 2017). Consistently, as displayed in Table 1, ICP has been documented to decrease with elevating the patient, whether by full-body tilt or elevating the head and thorax (Ryu et al., 2016). The ideal angle of elevation was shown to be 30° (Kim et al., 2017). Therefore ACD+ITD HUPCPR has the potential to improve patient outcomes, given its documented ability to reduce ICP in the porcine model of cardiac arrest. Left unanswered, however, is whether or not this method reduces ICP and raises CerPP in human OHCA significantly enough to improve survival to discharge and neurological outcome.

Furthermore, while ROSC rates appeared higher for subjects given ACD+ITD HUPCPR (as observed by Ryu et al., 2016 and Moore et al., 2017), differences in drug dosages, energy delivered during defibrillation, length of time in altered positions of HUP, SUP and HDT as well as timings of interventions between the studies render their findings difficult to compare with each other. They are also difficult to translate to OHCA as both the Resuscitation Council (UK) (2017) and American Heart Association (2015) recommend immediate defibrillation for OHCA when the patient is found in a shockable rhythm. This extreme heterogeneity and variance from standard treatment makes it difficult to determine when HUPCPR should be initiated during ALS to be of most benefit. Additionally, Kim et al. (2017) displayed ROSC for all subjects involved in their study, each of which experienced HUT, SUP and HDT positioning.

While the research analysed cannot answer the question set by this systematic review, it strongly suggests the next steps required to identify the potential benefits of augmented HUPCPR in OHCA, namely re-evaluating a translational cadaver model as initially attempted by Moore et al. (2018).

The novel approach demonstrated by Moore et al. (2018) to establishing a translational cadaver model to overcome the inherent physiological differences in vasculature between pigs and humans requires more robust investigation. The two main areas of improvement would be identifying a more realistic substitute for blood than saline (one of equal viscosity) and refraining from diverting the blood replacement from the extremities of the cadavers.

Furthermore, current pre-hospital resuscitation guidelines should be observed during the attempted drug resuscitation portion of the experiment to allow greater comparison to OHCA. For example, porcine subjects, left in induced VF and untreated for 6–7 minutes to represent a realistic OHCA without bystander CPR (time-frame in line with ambulance response times according to guidance from NHS England, 2017), should be subjected to immediate defibrillation, followed by initiation of augmented HUPCPR while following recognised resuscitation guidelines. Studies should only alter the time of initial defibrillation should ROSC be continually achieved prior to observation of augmented HUPCPR. This process would also help identify at which stage during resuscitation HUPCPR should be initiated, for the greatest benefit to patient survival discharge.

Building upon the study by Putzer et al. (2018), a further study into cerebral tissue oxygenation should be conducted but with augmented HUPCPR rather than C-HUPCPR. This would further evidence whether or not augmented HUPCPR increases CerPP to a great enough extent to improve cerebral tissue oxygenation and potentially improve neurological outcome post-ROSC.

Finally, should favourable ethical opinion be gained, it would be beneficial to facilitate an investigation to study the quality of neurological outcome of porcine subjects following successful resuscitation from augmented (ICD+ACD) HUPCPR by allowing subjects to recover and be observed for a set period of time. The International Liaison Committee on Resuscitation (ILCOR) (2015) states the Utstein criteria for assessment of successful resuscitation is quality of life measured at 12-month survival – this may be unrealistic at this early stage of clinical trials but it should influence the decision on what length of time the porcine subjects are re-evaluated. Also, as Ryu et al. (2016) demonstrated equal ROSC rate between augmented HUPCPR and augmented supine CPR, subject assessment at a suitable time post-ROSC would help establish the quality of ROSC achieved and elucidate the benefit of augmented HUPCPR against augmented supine CPR. While Moore et al. (2017) found increased ROSC in their heads-up group, without further research this discrepancy in results remains unexplained.

Should these studies identify a promising rise in CerPP, human trials may be the next logical step in the development of augmented HUPCPR.

### Limitations

The limitations of the data found cannot be understated. First, no human clinical trials were identified by this systematic review. Second, the attempt at creating a translational HC model by Moore et al. (2018) was thwarted by poor investigative technique. Third, the pool of data is almost exclusively from the same bank of authors, with acknowledged conflicts of interest rooted in finance and employment. Fourth, discrepancies between ALS procedure observed and heterogeneity of results make these papers harder to directly compare than one would have anticipated given the overlap in authorship across five of the research endeavours. These weaknesses mean that, however conclusively these papers identify that ACD+ITD HUPCPR reduces ICP and increases CerPP and CoPP in the porcine model of cardiac arrest, the results are difficult to extrapolate to OHCA patients and therefore this procedure cannot yet be recommended for implementation in paramedic practice.

## Conclusion

The question set by this systematic review remains unanswered. As such, augmented HUPCPR cannot be recommended for paramedic practice and current resuscitation guidelines must remain adhered to.

The results of the critical appraisal are worthy of further investigation. First, a more robust attempt at a translational cadaver model to help extrapolate results from the porcine model of cardiac arrest to human OHCA and second, if favourable ethical opinion can be obtained, research into neurological outcome of porcine subjects following augmented HUPCPR. Depending upon the results of this further research, studies on human population in OHCA may then be formulated to ascertain its potential benefit, if any, on survival to discharge and neurological outcome.

## Conflict of interest

None declared.

## Funding

None.
